# Epidemiological characteristics of mumps from 2004 to 2020 in Jiangsu, China: a flexible spatial and spatiotemporal analysis

**DOI:** 10.1017/S095026882200067X

**Published:** 2022-04-08

**Authors:** Mingma Li, Yuxiang Liu, Tao Yan, Chenghao Xue, Xiaoyue Zhu, Defu Yuan, Ran Hu, Li Liu, Zhiguo Wang, Yuanbao Liu, Bei Wang

**Affiliations:** 1 Key Laboratory of Environment Medicine Engineering of Ministry of Education, Department of Epidemiology and Health Statistics, Southeast University School of Public Health, Nanjing 210009, Jiangsu, China; 2 Department of Expanded Program on Immunization, Jiangsu Provincial Center for Disease Control and Prevention, Nanjing 210009, Jiangsu, China

**Keywords:** Clusters, flexible shaped scan statistics, mumps, spatial autocorrelation, spatial-temporal analysis

## Abstract

The mumps resurgence has frequently been reported around the world in recent years, especially in many counties mumps vaccines have been widely used. This study aimed to describe the spatial epidemiological characteristics of mumps in Jiangsu, and provide a scientific basis for the implementation and adjustment of strategies to prevent and control mumps. The epidemiological characteristics were described with ratio or proportion. Spatial autocorrelation, Tango's flexible spatial scan statistics, and Kulldorff's elliptic spatiotemporal scan statistics were applied to identify the spatial autocorrelation, detect hot and cold spots of mumps incidence, and aggregation areas. A total of 172 775 cases were reported from 2004 to 2020 in Jiangsu. The general trend of mumps incidence is declining with a bimodal seasonal distribution identified mainly in summer and winter, respectively. Children aged 5–10 years old are the main risk group. A migration trend of hot spots from southeast to northwest over time was found. Similar high-risk aggregations were detected in the northwestern parts through spatial-temporal analysis with the most likely cluster time frame around 2019. Local medical and health administrations should formulate and implement targeted health care policies and allocate health resources more appropriately corresponding to the epidemiological characteristics of mumps.

## Introduction

Mumps is a highly contagious disease of the respiratory system caused by the mumps virus [1, 2]. As a vaccine-preventable disease, the incidence and the number of serious complications due to mumps had been substantially reduced since the successful introduction of the mumps-containing vaccine (MuCV) in 1967 and the global high coverage implementation of two doses MMR (Measles, Mumps and Rubella) combined vaccine [3]. The epidemic situation of mumps in different countries and regions varies with diverse vaccination plans, including local vaccine coverage, age and times of vaccination [4–6]. In recent years, several mumps resurgences were reported in the United States [7], Australia [8], France [9], Britain [10] and other areas [11]. Most of the cases in these outbreaks were adolescents who had received two-dose MMR. Mumps is still a highly concerning public health issue with a severe disease burden.

According to the regional report of WHO, there were 129 120 cases of mumps reported in China, accounting for 47.97% of the global annual total reported cases [12]. MMR was introduced in the National Expanded Program on Immunization (EPI) by the Chinese government in 2007. Children aged 18–24 months can receive one dose MuCV [13]. Unexpectedly, no significant decline in the mumps epidemic was observed, whereas one dose MuCV coverage steadily increased [14]. From 2004 to 2018, more than 4 200 000 mumps cases were reported in China, with an annual average incidence of 21.44 per 100 000 [15]. To decrease the incidence of mumps, the 2-dose MMR schedule with the first dose at the age of 8 months and the second dose among 18–24 months, was carried out nationwide in China from July 2020. Jiangsu is an economically developed province in the middle east of China with a large number of migrants and complex climate features. Since the beginning of the 21st century, some small-scale mumps epidemics still often occurred in various parts of the province. In 2019, nearly 40 public health emergencies caused by mumps were reported in schools in Jiangsu. However, there has been no comprehensive description of the epidemiological pattern of mumps in Jiangsu as far as we know.

Geographical information systems (GIS), which can effectively use the spatial information of diseases to analyse the historical surveillance data and obtain the statistically significant clusters [16], have been widely applied to explore the distribution of various communicable diseases such as malaria [17], measles [18], COVID-19 [19] and some other fields [20, 21]. Flexible shaped scan statistics have higher detection efficiency for irregularly shaped aggregation areas and can obtain a more accurate estimation of the real situation [22, 23]. This improved flexible irregular scan statistic will determine a more explicit most likely aggregation region, thus further helping to reasonably allocate scarce health resources.

Therefore, to fill the knowledge gap and have a more comprehensive understanding of mumps, we conducted a systematic spatial scan analysis to identify the spatial and spatiotemporal epidemiological characteristics of mumps at the county level in Jiangsu from 2004 to 2020. Hence to provide a more scientific basis for further research on mumps, adjustment of strategies to prevent and control the mumps epidemics.

## Materials and methods

### Study area

**Fig. 1.**
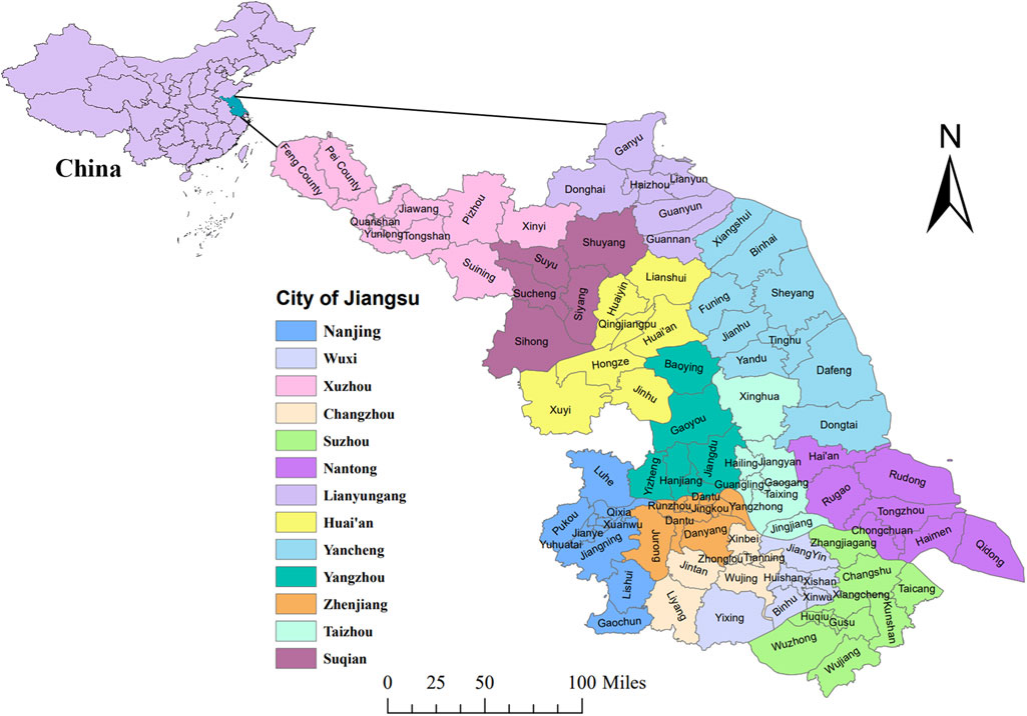
The geo-location and city/county distribution of Jiangsu Province in China. (The map was created with ArcGIS software version 10.8).

Jiangsu (30°45′-35°08′ N, 116°21′-121°56′ E), located in the eastern coastal area of mainland China, has a total land area of 107 200 km^2^, most of which are below 50 metres above sea level. Jiangsu is mainly dominated by plains and hills, crossed by the Yangtze River and east surrounded by the Yellow Sea. The special geographical location and terrain diversify its climate characteristics with an annual average temperature of 13.6–16.1°C. By the end of 2020, Jiangsu had an inhabitant population of about 84.75 million with 13 administrative cities and 95 districts or counties, all of which were included in this study and geographically shown in Figure 1. The map of China and Jiangsu was obtained from the National Catalogue Service for Geographic Information System and re-checked with the administrative divisions issued by Jiangsu Civil Affairs Bureau in 2020.

### Data collection and management

Data on the mumps cases between 2004 and 2020 were obtained from the China Information System for Disease Control and Prevention (CISDCP), which was established in 2004 to monitor legally reported infectious diseases covering all county/district level medical and public health institutions. All mumps patients were diagnosed with the ‘Diagnostic criteria for Mumps’ issued by the Chinese Ministry of Health [24], which is based on epidemiological exposure history, clinical manifestations and associated laboratory test positive results. Confirmed or suspected mumps cases must be reported through the network or an infectious disease report card by doctors within 24 h and then assessed by trained administrators. The data exported from the CISDCP were managed with Microsoft Excel 2019, and a unique administrative code was used to match cases to the county-level polygons vector map of Jiangsu.

### Descriptive epidemiological analysis

The basic epidemiological characteristics include gender, occupational classification, age and seasonality of cases were described with numbers, ratios or proportions. The annual incidence of mumps was calculated by dividing the annual number of reported mumps cases by the annual population (* 100 000). We also calculated the average annual incidence over the whole study period by dividing the average annual number of mumps cases by the average population (* 100 000).

In order to better comprehend and visualise the geographical distribution of mumps cases, we drew the annual incidence rates map of mumps at the county/district level with ArcGIS 10.8. This spatial map shows the distribution of mumps incidence in different years more directly and intuitively and counts the number of regions under specific groups (6 groups in this study) of incidence rate, which can help to guide further analysis of overall trends.

### Spatial autocorrelation analysis

Spatial autocorrelation analysis is a crucial method to test whether the observed values of the same variable are significantly correlated at a location with its surrounding positions, and identify potential clusters regions in the study area. Considering the data of mumps incidence at the county level are low-value data in a small area, there may be some instability and random error using direct analysis. Referring to previous studies [21, 25], we used spatial empirical Bayesian (SEB) to smooth the mumps incidence with a spatial matrix weights file generated by the algorithm of K-Nearest neighbours to improve the stability and reliability of the results. The SEB model made appropriate adjustments to the incidence of irregular counties with small area and more adjacent points while having less impact on the large counties.

Global spatial autocorrelation was used to detect the spatial dependence of mumps incidence in a global area through the global Moran's index (Moran's *I*). The Moran's *I* range from −1 to +1, and the |*I*| value closer to 1 indicates a stronger autocorrelation. If *I* > 0 with statistical significance (*P*-value <0.05 and |*Z*-scores| <1.96) represents positive spatial autocorrelation, reversely, shows a negative spatial autocorrelation. An *I* = 0 indicates no spatial autocorrelation exists, which means that the distribution of mumps in Jiangsu is random and there is no statistical cluster [26]. Local indicators of spatial autocorrelation (LISA) reflect four specific spatial patterns through local spatial autocorrelation, and can effectively detect the spatial difference of mumps incidence caused by the small area at the county level. Four different cluster patterns are high-high (high-incidence regions surrounded by high-incidence regions); high-low (high-incidence regions surrounded by low-incidence regions); low-high (low-incidence regions surrounded by high-incidence regions; and low-low (low-incidence regions surrounded by low-incidence regions) [27]. We used GeoDa software version 1.20 to analyse all spatial autocorrelation of mumps incidence, which were smoothed by a specific spatial matrix weights file.

### Spatial and spatiotemporal irregular scan analyses

The correlation pattern of mumps incidence at the county level was realised through spatial autocorrelation, but limited to the comparison of various counties within a single year, which made it difficult to identify the aggregation areas during the entire study period. Therefore, we further adopt purely spatial scan analysis to detect irregular clusters of mumps by Tango's flexible spatial scan statistics [22, 23] in FleXScan v3.1.2, which is based on a Poisson model with a restricted log-likelihood ratio (rLLR, default alpha = 0.2). The maximum spatial cluster size and Monte Carlo replications were set to 15 and 999, respectively.

Kulldorff's retrospective space-time scan statistics [28] were defined by a cylindrical window with an elliptic space base and with height corresponding to the time in this study. The window moved in space and time dimension simultaneously for each possible location and time frame, then automatically calculating the relative risk (RR) and log-likelihood ratio (LLR) for each potential cluster with a discrete Poisson model. The RR was calculated from the ratio of the observed cases to the expected cases inside and outside the window. The most likely cluster was defined with the largest LLR, which compared the observed incidence with the expected incidence to identify specific clusters, and the *P*-value of LLR was obtained from 999 times Monte Carlo simulation. Referred to previous research [21, 29] and times tests of different parameters, we finally set the maximum spatial cluster size to 15% of the population at risk, the maximum and minimum temporal clusters size to 20% of the timeframe and one month, respectively. The spatiotemporal analysis was carried out in SaTScan v10.0.1 software, and its results were visualised by ArcGIS 10.8 (ESRI, Redlands, CA, USA). *P* < 0.05 for both sides was considered statistically significant in this study.

## Results

### General epidemiological characteristics

**Fig. 2.**
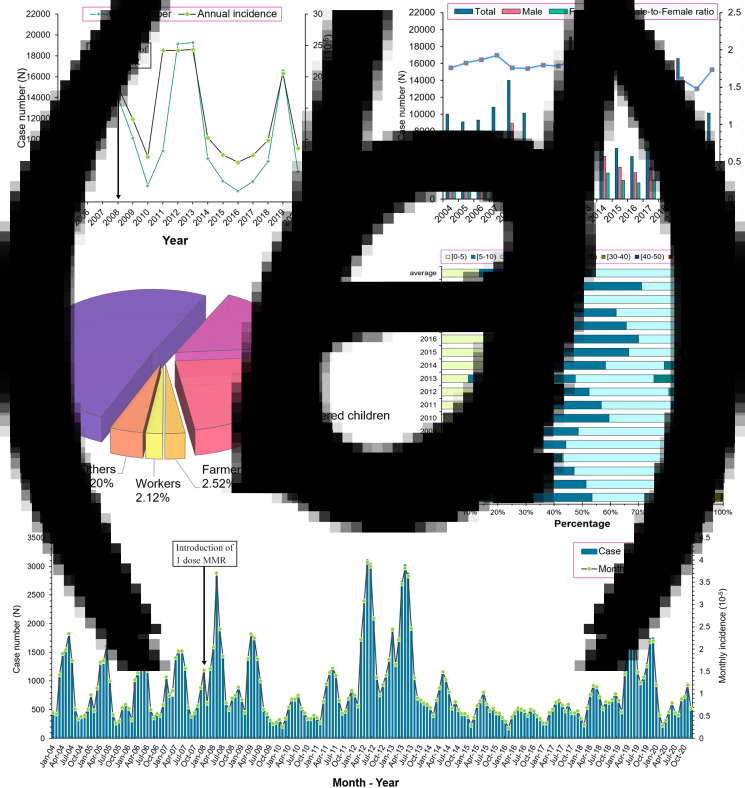
Epidemiological characteristics of mumps in Jiangsu from 2004–2020. (a) The annual case number and incidence of mumps; (b) The annual number of mumps cases in different gender; (c) The population and occupational distribution of mumps; (d) The age distribution of mumps; (e) The monthly incidence distribution of mumps.

There were 172 775 mumps cases reported in Jiangsu Province from 2004 to 2020 with an annual average incidence of 12.99 per 100 000 (ranging from 6.32 per 100 000 in 2016 to 24.34 per 100 000 in 2013). The male and female cases were 109 585 and 63 190, respectively, with an average male-to-female ratio of 1.73 (ranging from 1.47 in 2020 to 1.93 in 2007). The annual incidence and yearly case numbers are shown in Figures 2a and 2b. The fluctuating trend of mumps incidence (per 100 000) in Jiangsu can be divided into several representative stages. Firstly, it descended slightly from 13.25 in 2004 and then continuously climbed to 18.37 in 2008. Next, it dropped to 7.19 in 2010 and rapidly increased to 24.22 in 2011 with a peak period lasting until the end of 2013. A low level of incidence with slight range fluctuation was maintained from 2014 to 2018, but the incidence rose to 20.55 in 2019 again and dramatically declined to 8.58 in 2020.

Regarding the occupational distribution displayed in Figure 2c, students (50.96%) were the leading group at risk of mumps, followed by scattered children (23.77%) and childcare children (16.43%). Children aged 5–9 (39.72%) years old accounted for the highest proportion of the total cases, followed by cases aged 10–14 (29.44%) and 0–4 (13.46%) years old (Fig. 2d). Furthermore, a significant bimodal seasonality of mumps can be observed in the monthly incidence distribution (Fig. 2e). The primary peak occurred from April to July, whereas the second one was from October to January in the next year, which was similar among different years.

### Geographical distribution

**Fig. 3.**
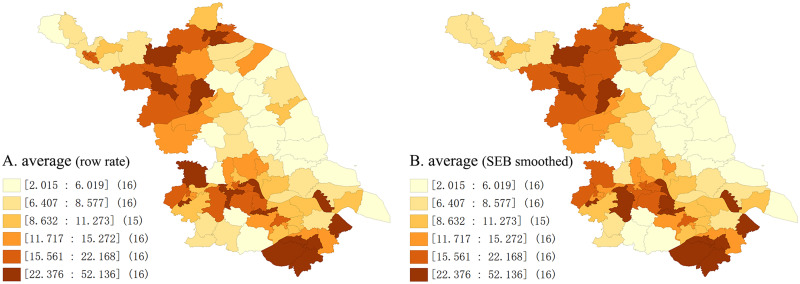
Average annual incidence at county/district level in Jiangsu from 2004 to 2020. (A) The raw rate of average annual incidence (/100 000); (B) The spatial empirical Bayesian smoothed average annual incidence (/100 000). ([incidence] (county numbers)).

**Fig. 4.**
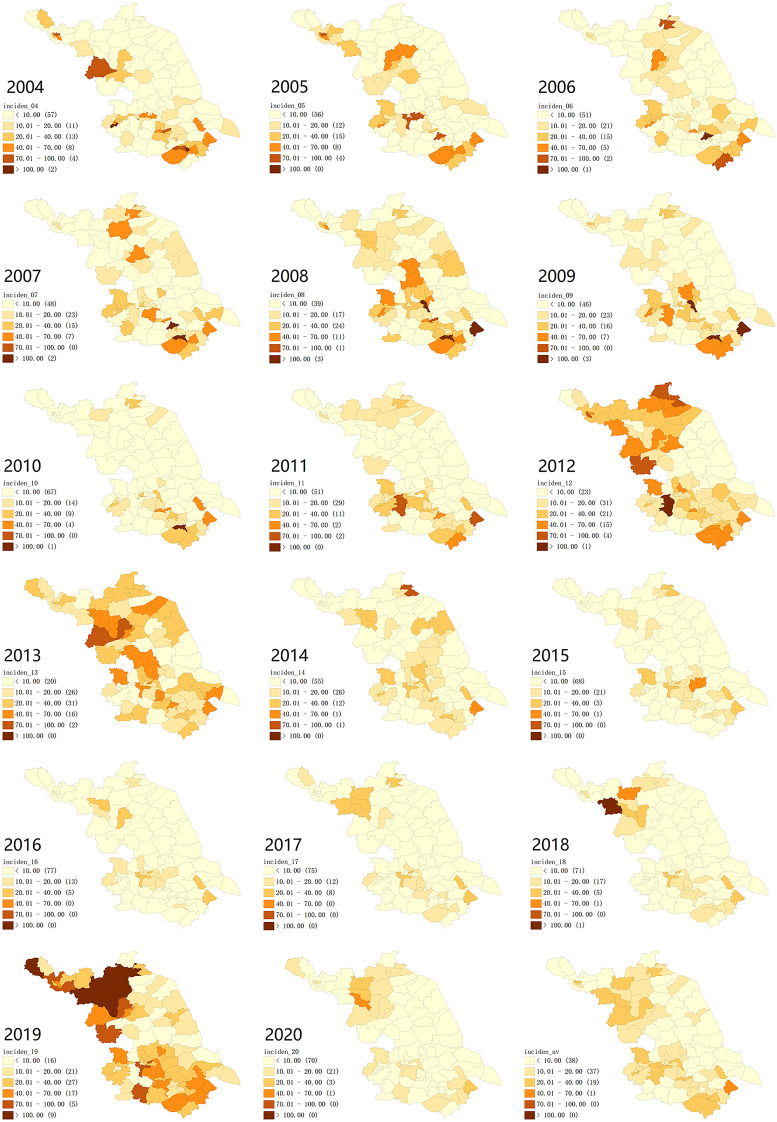
The annual incidence map of mumps in Jiangsu from 2004 to 2020.

The average annual incidence map at the county level (Fig. 3) indicated that the high incidence counties of mumps mainly concentrated in the northern and southern parts of Jiangsu. The cities of Lianyungang, Suqian, Nanjing, Zhenjiang and Suzhou contributed a majority of high-incidence counties throughout the whole study period. Except for several small areas with multi-neighbours, there was no distinctive difference can be found in the SEB smoothed average annual incidence compared with the raw rate, and both of the general geographical patterns are similar. The map of annual incidence from 2004 to 2020 (Fig. 4) shows that the locations of the high incidence counties were constantly transferring, and it can be roughly seen that the migration trend of the high-incidence counties was from the southeast to northwest of Jiangsu over time. Moreover, the middle eastern parts of Jiangsu retained a relatively low mumps incidence during the observation period.

### Spatial autocorrelation analysis

The results of the global spatial autocorrelation analysis listed in Table 1 demonstrate that there was a significant positive spatial autocorrelation in Jiangsu from 2004 to 2020. The annual Moran's *I* also indicated a similar statistically significant positive spatial autocorrelation in the yearly analysis, except in 2007, 2008, 2012 and 2014.

**Table 1.** The global spatial autocorrelation of mumps in Jiangsu from 2004 to 2020

Year	Moran *I*	*Z-*value	*E*[*I*]	Mean	s.d.	*P-*value
2004	0.151	3.116	−0.011	−0.009	0.052	0.005
2005	0.104	2.195	−0.011	−0.011	0.052	0.030
2006	0.117	2.520	−0.011	−0.009	0.050	0.130
2007	0.037	1.096	−0.011	−0.011	0.044	0.100
2008	0.029	0.879	−0.011	−0.011	0.046	0.167
2009	0.109	2.408	−0.011	−0.013	0.051	0.025
2010	0.242	4.840	−0.011	−0.010	0.052	0.001
2011	0.137	2.850	−0.011	−0.011	0.052	0.010
2012	0.118	1.970	−0.011	−0.011	0.050	0.035
2013	0.194	3.858	−0.011	−0.009	0.052	0.002
2014	−0.104	0.013	−0.011	−0.011	0.049	0.463
2015	0.183	3.950	−0.011	−0.011	0.049	0.002
2016	0.118	2.560	−0.011	−0.014	0.051	0.013
2017	0.161	3.244	−0.011	−0.011	0.053	0.004
2018	0.152	4.270	−0.011	−0.011	0.038	0.002
2019	0.292	7.956	−0.011	−0.010	0.038	0.001
2020	0.338	7.165	−0.011	−0.010	0.049	0.001
Average	0.147	3.030	−0.011	−0.013	0.053	0.004

*Note: E*[*I*]: the theoretical mean of Moran's *I* statistic. Mean and s.d.: the centralised and discrete trends of simulated empirical distribution.

**Fig. 5.**
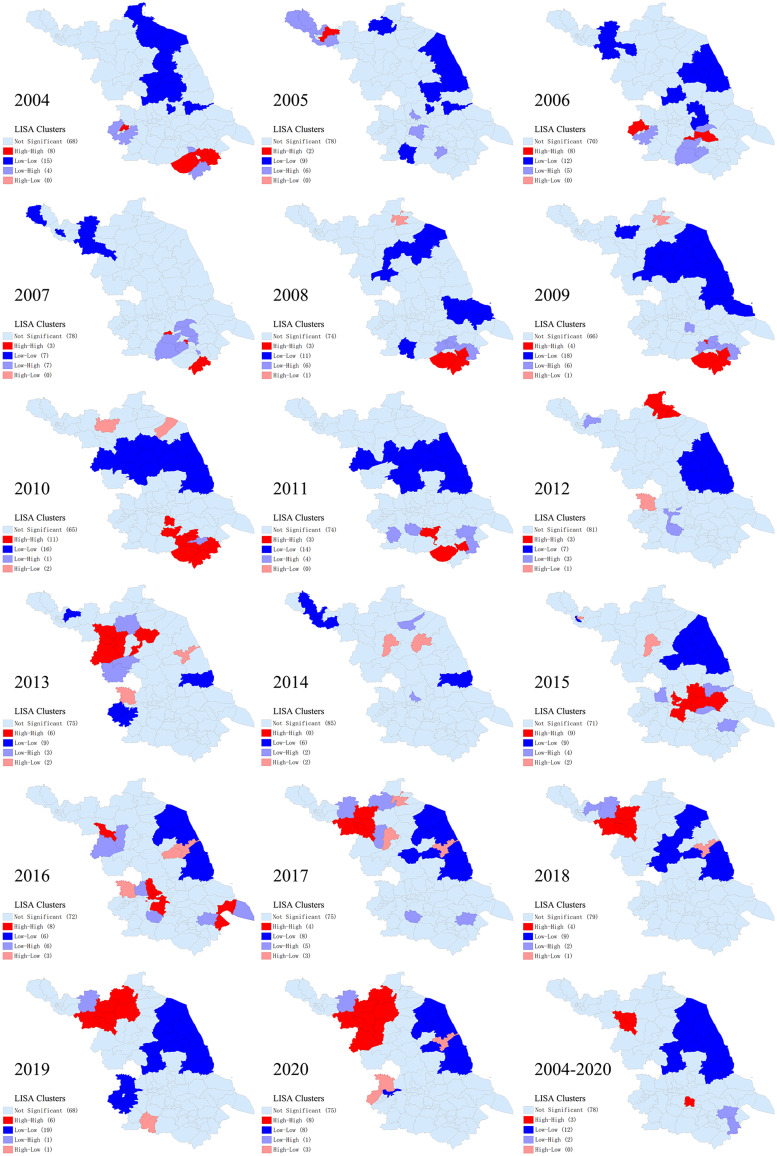
Yearly LISA clusters maps for mumps incidence in Jiangsu from 2004 to 2020.

The LISA analysis of the average annual incidence detected 4 hot spots (High-High) and 12 cold spot counties (Low-Low) for mumps in Jiangsu, and the yearly cluster pattern of LISA is presented in Figure 5. 2010 was the year with the most hotspots of 11 counties, as listed in Table 2. There was no hot spot detected in 2014. Cold spots were stably identified in the middle eastern parts of Jiangsu. The transfer tendency of hot spot clusters from 2004 to 2020 is from the southeast to the northwest of Jiangsu, similar to the changing trend of incidence rate distribution in Figure 4.

**Table 2.** Hot spots lists resulting from the local indicators of spatial analysis from 2004 to 2020

Year	Counties (n)	Hot spots of High-High aggregation areas
2004	8	Xuanwu, Qinhuai, Jianye, Nanjin Gulou, Huqiu, Wuzhong, Xiangcheng, Kunshan
2005	1	Jiawang
2006	9	Xuanwu, Qinhuai, Jianye, Nanjin Gulou, Pukou, Jiangyin, Tianning, Zhonglou, Kunshan
2007	4	Liangxi, ZhongLou, Wujiang, Wuzhong
2008	3	Wuzhong, Gusu, Wujiang
2009	4	Liangxi, Wuzhong, Gusu, Wujiang
2010	11	Xishan, Binhu, Liangxi, Xinwu, Xinbei, Wujin, Huqiu, Wuzhong, Gusu, Wujiang, Kunshan
2011	4	Binhu, Wujin, Wuzhong, Gusu
2012	3	Lianyun, Haizhou, Ganyu
2013	7	Qingjiangpu, Huai'an, Lianshui, Sucheng, Suyu, Siyang, Sihong
2014	0	none
2015	9	Rugao, Guangling, Jingkou, Danyang, Yangzhong, Hailing, Gaogang, Jiangyan, Taixing
2016	10	Xinbei, Taicang, Haimen, Guangling, Hanjiang, Jingkou, Runzhou, Danyang, Sucheng, Yangzhong
2017	4	Suining, Xinyi, Sucheng, Suyu
2018	4	Suining, Xinyi, Sucheng, Suyu
2019	6	Suining, Xinyi, Donghai, Sucheng, Suyu, Shuyang
2020	8	Suining, Xinyi, Donghai, Sucheng, Suyu, Shuyang, Siyang, Sihong
Average	4	Xinbei, Sucheng, Suyu, Siyang

### Spatial and spatiotemporal irregular clusters

**Fig. 6.**
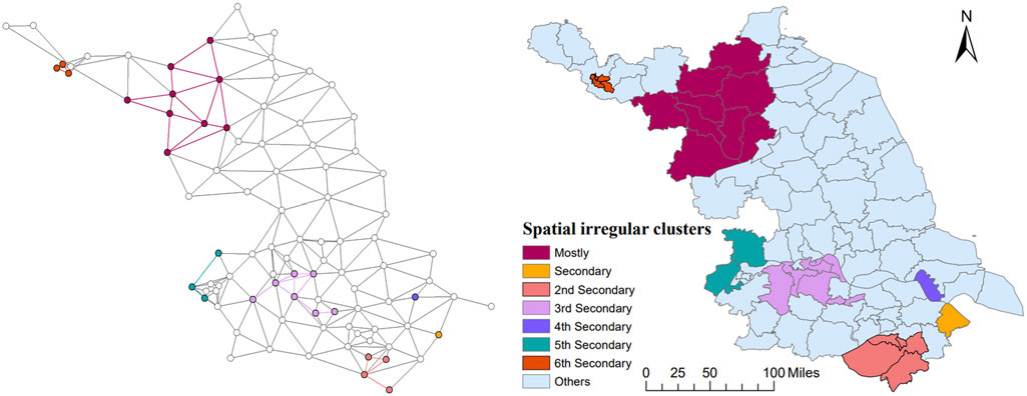
The pure spatial clusters of mumps in Jiangsu from 2004 to 2020. (Clusters detected by FleXScan v3.1.2 with Tango's flexible scan statistics (left), and visualised in different colours through ArcGIS 10.8 (right)).

Based on the average cases and average population, Tango's flexible spatial scan statistics indicated that the distribution of mumps in Jiangsu was not random in space between 2004 and 2020. A total of 13 spatial clusters were detected and seven of them with statistical significance are listed in Table 3. The most likely cluster was identified with rLLR and RR values 284.36 and 1.76, respectively. Located in the northwestern part of Jiangsu, the mostly cluster (radius: 153.11 km) consisted of Donghai (belonging to Lianyungang City), Xinyi and Suining (Xuzhou City), and other six counties including the entire city of Suqian. This result suggests that the mumps control measures in these counties should receive more attention. The geographical distribution of the remaining six clusters is visualised in Figure 6.

**Table 3.** The spatial irregular clusters of mumps detected by Tango's flexible scan statistics

Cluster type	Counties (n)	Radius (km)	Observed cases	Expected cases	rLLR	RR	*P* value
Most likely	9	153.11	1851	1050.48	284.36	1.76	0.001
Secondary	1	0^a^	332	82.84	214.85	4.01	0.001
2nd secondary	4	42.46	972	505.52	180.46	1.92	0.001
3rd secondary	7	82.30	808	479.91	98.48	1.68	0.001
4th secondary	1	0^a^	282	137.47	59.14	2.05	0.001
5th secondary	3	52.51	431	246.12	58.34	1.75	0.001
6th secondary	3	12.92	263	172.40	20.89	1.53	0.001

a *Note:* Radius = 0 means there is only one county in this cluster.

**Fig. 7.**
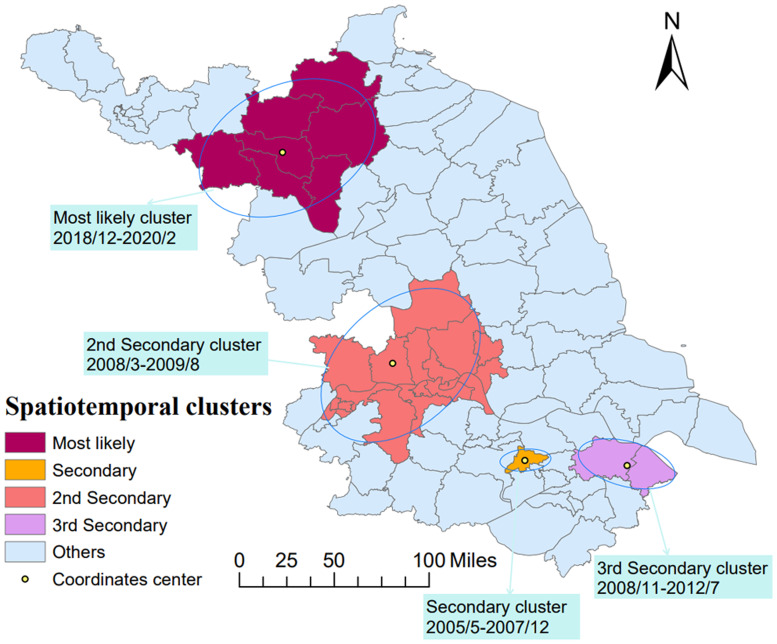
The space-temporal clusters of mumps in Jiangsu from 2004 to 2020.

Kulldorff's spatiotemporal scan analysis detected four irregular high-incidence clusters of mumps from 2004 to 2020, including a total of 28 counties in Jiangsu. The detailed information of the spatiotemporal cluster is reported in Table 4 and visualised in different colours in Figure 7. The most likely spatiotemporal cluster was in a location similar to the first purely spatial scan cluster, covering seven counties (coordinates centre: 118.36 E, 34.00 N) from December 2018 to February 2020 (LLR = 11 900.72, RR = 8.69). The 2nd secondary, secondary and 3rd secondary cluster was identified in the centre and southern parts of Jiangsu, respectively.

**Table 4.** The spatial-temporal irregular clusters of mumps cases from 2004 to 2020

Cluster type	Counties (n)	Time frame	Coordinates centre	Observed cases	Expected cases	LLR	RR	*P* value
Most likely	7	2018/12 – 2020/2	118.36 E, 34.00 N	9481	1146.05	11900.72	8.69	0.001
Secondary	1	2005/5 – 2007/12	120.21 E, 31.65 N	3012	212.05	5215.04	14.44	0.001
2nd secondary	18	2008/3 – 2009/8	119.20 E, 32.39 N	6465	2002.95	3171.44	3.31	0.001
3rd secondary	2	2008/11 – 2012/7	120.99 E, 31.61 N	3282	671.25	2617.61	4.96	0.001

## Discussion

This study, for the first time, systematically described the basic epidemiology and clarified the spatiotemporal distribution characteristics of mumps in Jiangsu using an improved spatial scan statistic. During the entire study period, results revealed that the number of yearly reported cases fluctuated obviously but decreased generally, from 10 028 in 2004 (13.25 per 100 000) to 6925 in 2020 (8.58 per 100 000). Several mumps outbreaks were identified in some cities during the different time frames. Before the MMR vaccine was included in the EPI in 2008, mumps vaccination coverage was very low. The incidence of mumps in Jiangsu Province showed a slow climbing trend from 2004 to 2008 and was controlled with the introduction of MMR, which was similar to what was witnessed in the whole country and several other provinces during 2009–2010 [15, 29]. However, mumps incidence has a sharp increase from 2011 to 2013, the possible reasons might be the accumulation of potentially susceptible populations who missed vaccination for various reasons and the limited effect of the one dose MMR on the mumps epidemics. There are also other plausible explanations for the unexpected outbreak in vaccinated-population explored by previous research studies: waning immunity; the persistence of antibodies with time after vaccination; the efficacy of mumps vaccine, varied according to the doses of vaccinations and the genotypic differences between vaccine strains and wild virus strains [9, 30]. The incidence of mumps remained at a relatively low level between 2014 and 2018, this decrease may not be attributed to the effect of the vaccine, but the vast majority of susceptible people naturally infected and gained resistance during the 2011–2013 pandemic. At the same time, people have no chance to gain enough immunity through natural infection under the background of vaccination, and the susceptible people in the population are accumulating, so another outbreak occurred in 2019. The whole trend shows that the incidence peak of mumps occurs every 5 years, and one dose of MMR vaccination does not seem to have a significant effect on the prevention of mumps. It should be noted that the cases of mumps reported in 2020 might be less than the actual number of cases due to the outbreak of COVID-19, the restrictions on population mobility and widespread use of masks have greatly reduced the risk of infection. These above results indicated that the introduction of two doses MMR to children is essential and is an effective strategy for mumps prevention and control, which was also been proven by many countries where two dose MMR vaccines were offered to the population at the child age. By July 2020, children can be vaccinated with two doses of MMR nationwide in China, but the specific effect still needs further observation.

Children aged 5–10 years old took up most of the mumps cases during the study period (Figs 2c and 2d), which is consistent with previous study findings. Because these children had only been vaccinated one dose MMR, the mumps specific antibody titre may be waned below the protective level, hence making them more prone to mumps infection. Additionally, kindergartens or primary schools may facilitate the spread of mumps virus because of overcrowding and personal hygiene habits. Since 2014, the increasing proportion of mumps in elderly over 50 years old was also be observed (Fig. 2d). It might be attributed that the elderly population missed the opportunity of vaccination and had no chance to obtain natural infection immunity from the decreasing mumps epidemic. Moreover, fragile health situation also makes the elderly more vulnerable to the virus. Based on the findings of the high-risk population and the limited efficacy of the MMR, more supplementary immunisation targeting the children and the elderly are needed.

Mumps has distinctive seasonal characteristics as a respiratory infectious disease, our study also found bimodal seasonal distribution results similar to the previous studies [21, 29, 31], with the first peak occurred between April to July, and another small one from October to January in the next year (Fig. 2e). On the one hand, it may be related to higher population mobility in summer (people prefer to go out this season) and higher vulnerability in winter. On the other hand, it may correlate to some climate factors. Qiongying Yang's research team [32] found that mean temperature, relative humidity, wind velocity and atmospheric pressure might be important predictors of the mumps incidence. A study in Jining [33] reported a linear relationship of mean temperature, relative humidity, sunshine duration and daily wind speed with mumps incidence when exceeding a certain threshold. Another study [34] found a non-linear relationship between mumps and temperature in Shandong, and analogical correlations were identified in some other studies in China [35–37]. Nonetheless, no similar research has been carried out to analyse the correlation of mumps incidence and meteorological factors in Jiangsu, the potential association still can be summarised by the distribution map of mumps incidence and spatial autocorrelation results in this study. The eastern parts of Jiangsu are close to the Yellow Sea, which brings more wind and other possible protective meteorological conditions against mumps. Therefore, this region has maintained a lower incidence rate during the observation period (Fig. 4). Simultaneously, the results of LISA clusters also reveal that there are always statistically significant cold spots (Low-Low) around this area (Fig. 5). These seasonal characteristics and meteorological factors should be more considered to formulate and implement targeted interventions for the prevention and control of mumps.

The results of spatial analysis in this study show a significant spatial autocorrelation in the incidence distribution of mumps in Jiangsu, indicating that the onset of mumps in some counties would affect their adjacent counties or districts relatively (Table 1). The changing hot spots in the yearly LISA clusters also indicate the regional instability of mumps (Fig. 5). From 2004 to 2020, the transfer trend of hot spots from southeast to northwest may be related to the development of local economic and health care services. The most likely aggregation cluster was detected in the inland area of northwest Jiangsu by Tango's flexible purely spatial scan statistics, with the majority counties located in the cities of Suqian, Xuzhou and, Lianyungang (Table 3 and Fig. 6). As is known to all, the economy of northwestern Jiangsu is not as developed as that of the central and southern regions, so it may be relatively inadequate in terms of resource support and health services. In addition, the effectiveness of single-dose MMR vaccine is not so ideal. Several studies have reported that the protective effect of MuCV diminishes over time and declines rapidly by the 5th year after vaccination [38, 39]. It may be the comprehensive reasons of these economic, meteorological and, vaccine factors that explain the northward movement of mumps hotspot clusters. Consequently, medical and health institutions in these high-risk areas are suggested to improve local health care services through increasing the vaccination coverage of two or three doses of MuCV and paying more attention to the risk population of mumps like school-age children and the elderly.

The location of the four spatiotemporal aggregations identified by elliptical window scanning is actually consistent with the distribution feature of the results of LISA and purely spatial scanning analyses (Fig. 7). The most likely cluster is located in the northwestern of Jiangsu, which is similar to the pure spatial aggregation, corresponding to the outbreak around 2019. The other three statistical significantly clusters distributed in the south of Jiangsu during the earlier time frame of the study period. This outcome proves that the model selection and parameter setting we carried out in different analyses in this study are reasonable in another way.

Several obvious advantages can be pointed out in this study. First of all, compared with the traditional methods of epidemiological analysis, spatial-temporal analysis can better visualise the distribution of disease and detect whether ‘active’ aggregations are forming [26] in this study. For example, LISA has unique advantages in detecting true aggregations with low false-positive rates especially performing well on outlier detection [27]. For further improvements over previous studies, we selected flexible scanning windows in both purely spatial and spatiotemporal scanning, to get higher detection efficiency for irregularly shaped aggregations. Additionally, based on the announcement emphasised by Tango, restricted-log likelihood rate (rLLR) was applied to identify statistically significant clusters in this study, which can get a more accurate estimation [22, 40]. In the meantime, several insurmountable limitations need to be discussed. Firstly, the number of mumps cases may be inaccurate due to the similar clinical features with other respiratory system diseases, and the passive monitoring mechanism of CISDCP. Secondly, this study mainly focused on the epidemiological characteristics of mumps and described it through the methods of spatial analysis. However, the relationships between mumps and meteorological parameters, sociological factors and vaccine efficacy still need further investigation and evidence.

## Conclusions

This study applied flexible scan statistics to systematically describe the epidemiological and spatiotemporal features of mumps in Jiangsu from 2004 to 2020 and determined the vulnerable population and high-risk areas of mumps. Despite the overall incidence trend is declining, the outbreak of mumps is still possible. School-age children at 5–10 years old are more susceptible to mumps, and the elderly over 50 are also at high risk. At present, the epidemic of mumps may mainly occur in counties located in relatively poor regions in the northwest and cities with high population density. Therefore, targeted health care policies should consider vulnerable groups and high-risk areas, properly allocate health resources considering the seasonal characteristics of mumps and local meteorological factors and improve the coverage and timeliness of MuCV.

## Data Availability

The data that support the findings of this study are available from the CISDCP but restrictions apply to the availability of these data, which were used under licence for the current study, and so are not publicly available. Data are however available from the authors under reasonable request and with permission of the Jiangsu Provincial Center for Disease Control and Prevention.

## Fundings

This research was supported by the National Natural Science Foundation of China (grant No. 82073623, 81502860) and the Jiangsu Provincial Medical Youth Talent Project (grant No. QNRC2016547), and the Medical and Scientific Research Project of Jiangsu Provincial Health and Family Planning Commission (grant No. H2017016). All of them had no role in the design of the study and collection, analysis, interpretation of data and in writing the manuscript.
